# Neuroimaging assessment of early and late neurobiological sequelae of traumatic brain injury: implications for CTE

**DOI:** 10.3389/fnins.2015.00334

**Published:** 2015-09-24

**Authors:** Mark Sundman, P. Murali Doraiswamy, Rajendra A. Morey

**Affiliations:** ^1^Duke-UNC Brain Imaging and Analysis Center, Duke University Medical CenterDurham, NC, USA; ^2^Department of Psychiatry, Duke University Medical CenterDurham, NC, USA; ^3^Duke Institute for Brain Sciences, Duke University Medical CenterDurham, NC, USA

**Keywords:** Traumatic brain injury (TBI), Tau, Chronic Traumatic Encephalopathy (CTE), neuroimaging, concussion, Positron Emission Tomography (PET)

## Abstract

Traumatic brain injury (TBI) has been increasingly accepted as a major external risk factor for neurodegenerative morbidity and mortality. Recent evidence indicates that the resultant chronic neurobiological sequelae following head trauma may, at least in part, contribute to a pathologically distinct disease known as Chronic Traumatic Encephalopathy (CTE). The clinical manifestation of CTE is variable, but the symptoms of this progressive disease include impaired memory and cognition, affective disorders (i.e., impulsivity, aggression, depression, suicidality, etc.), and diminished motor control. Notably, mounting evidence suggests that the pathology contributing to CTE may be caused by repetitive exposure to subconcussive hits to the head, even in those with no history of a clinically evident head injury. Given the millions of athletes and military personnel with potential exposure to repetitive subconcussive insults and TBI, CTE represents an important public health issue. However, the incidence rates and pathological mechanisms are still largely unknown, primarily due to the fact that there is no *in vivo* diagnostic tool. The primary objective of this manuscript is to address this limitation and discuss potential neuroimaging modalities that may be capable of diagnosing CTE *in vivo* through the detection of tau and other known pathological features. Additionally, we will discuss the challenges of TBI research, outline the known pathology of CTE (with an emphasis on Tau), review current neuroimaging modalities to assess the potential routes for *in vivo* diagnosis, and discuss the future directions of CTE research.

## Introduction

There has been a recent explosion of research focused on improving the diagnosis and treatment of Traumatic Brain Injury (TBI). The discourse has shifted to potential long-term effects following recent evidence in high-profile professional athletes linking head injuries with Chronic Traumatic Encephalopathy (CTE). The general population has gained a better understanding of TBI with heightened public awareness spurred, in large measure, by these high-profile cases. Meanwhile the medical community has made a concentrated effort on discovery with new research initiatives. However, with this heightened attention and national media coverage it is important that discussions carefully delineate evidence confirmed by empirical studies from hypotheses about this neurodegenerative disease process and its potential link to head trauma (DeKosky et al., 2010). Given the human brain's vast complexity and seemingly endless intricacies, it is of little surprise that many unknowns remain about TBI.

### The challenging landscape of TBI research

The challenge with investigating TBI begins with its definition (Tator, 2014). There are major differences in how various organizations define and classify head injuries, which have obscured treatment and research (Ruff et al., 2009). For instance, within a single panel of experts, The International Conference for Concussion in Sport, the operational definition of TBI has changed each of the four times the panel has met over the past decade (Aubry et al., 2002; McCrory et al., 2005, 2009). The definition of TBI has not kept pace with our rapidly evolving understanding of the injury and thus, the diagnosis of TBI remains vague. For the purposes of this article, the term concussion will be used interchangeably with mild TBI (mTBI), defined as a closed brain injury that results in specific clinical symptoms, generally from self-report, such as altered sensorium and loss of consciousness.

Amidst the ambiguity surrounding TBI and its definition, the data available indicates it is prevalent in all segments of society and is escalating in frequency. In the CDC's most recent report, TBI was referred to as a “silent epidemic” for it is widespread and often undetectable sequelae (Faul et al., 2010). TBI is currently the leading cause of morbidity and mortality worldwide in individuals under the age of 45 (World Health Organization, 2006). The World Health Organization (WHO) reports an estimated 10 million people suffer TBIs annually and predicts that TBI will be the third largest contributor to the global burden of disease and mortality by 2020 (World Health Organization, 2006). According to the CDC, falls are the most frequent cause for TBI accounting for roughly 35% of head injuries (Faul et al., 2010). People over 60 are at greater risk for these falls and, therefore, TBIs can be expected to increase given that this demographic group is projected to grow 223% between 1970 and 2025 to totaling 1.2 billion (Kalache and Gatti, 2003). The WHO reports that vehicular accidents are occurring at higher rates around the world as developing nations continue to adopt motorized forms of transportation that lack adequate regulations, safe roads, and safe equipment (World Health Organization, 2006). Recent estimates also indicate that recreational sports account for 1.6–3.8 million TBI each year in the United States alone (Langlois et al., 2005), a number that is up from 306,000 in 1991 (Thurman et al., 1998). Additionally, a higher-percentage of soldiers are experiencing TBI as new innovations in medicine (e.g., craniectomies, control of hemorrhagic bleeding) and their body armor make it more likely to survive injuries that would have previously proved fatal without adequately protecting their head (Okie, 2005). Laboratory studies of animals are helping to better understand the biomechanical forces associated with blasts, but more research is required to better understand the acute and chronic effects of these events (Cernak et al., 2011; Rubovitch et al., 2011; Wang et al., 2011; Goldstein et al., 2012; Xiong et al., 2013). Head injuries are so prevalent amongst this population that mTBI has been called the signature injury of the recent wars in Iraq and Afghanistan with an estimated 10–20% of all soldiers sustaining such injuries (Hoge et al., 2008; Elder and Cristian, 2009).

Despite their growing prevalence, concussions suffer a problem of perception; they are invisible injuries with no overt signs (e.g., surface wounds, broken bones) and patients rarely associate their symptoms with the traumatic incident making it exceedingly difficult for physicians to objectively measure (Nowinski, 2013). The subjective and equivocal nature of TBI often leads to misdiagnosis. Currently, clinicians diagnose concussions based on self-report of chronic symptoms that may include headache, nausea, vomiting, dizziness, fatigue, abnormal sleeping patterns, drowsiness, impaired memory/cognition, and more (McCrory et al., 2009; Randolph and Kirkwood, 2009; Randolph et al., 2009). Additionally, the clinical diagnosis relies on self-report of acute symptoms from patients, which is inherently unreliable (Delaney et al., 2002; McCrea et al., 2004). Though there is considerable debate and the exact prevalence is not entirely understood, approximately 10–15% of those diagnosed with concussions go on to develop post-concussive syndrome (PCS) where these symptoms persist for at least 3 months following the injury (Bigler, 2008; Williams et al., 2010). Additionally, there is often a delayed manifestation of symptoms, which leaves the patient less likely to associate their symptoms with the injury and omit medical care (Roozenbeek et al., 2013).

It has been widely reported that a significant portion of those suffering head injuries do not seek medical attention because (1) patients are unaware of the symptoms constitute a TBI or, (2) disincentives for reporting symptoms leads patients to intentionally conceal the incident to remain engaged in their activity (e.g., sports, combat) (Langlois et al., 2005, 2006). Experts have explored the utility of neuroimaging techniques to assist in diagnosis, but a reliable diagnostic imaging biomarker for mTBI remains elusive. Physicians may use conventional Magnetic Resonance Imaging (MRI) or Computed Tomography (CT) scans at the time of clinical assessment, but the Diffuse Axonal Injuries (DAI) often present in mild TBI are undetectable by these techniques (Shenton et al., 2012). The integration of kinematic data from accelerometers to assist with diagnosis has also been explored, but this practice has proven to be inadequate to date (Guskiewicz and Mihalik, 2011).

These limitations and challenges in the diagnosis of head injuries are problematic at a number of levels. First, those who experience neurological damage following trauma to the head may be in serious danger of second impact syndrome, which remains controversial in its own right due to its low incidence and the imperfect measures discussed above to identify the primary injury required to precede it (Bey and Ostick, 2009). Additionally, it is well established that those with a history of TBI are more susceptible to future injuries (Guskiewicz et al., 2000; Abrahams et al., 2014) and, therefore, should alter their behaviors or be observed with greater scrutiny if they choose to continue any activity that may presents a risk for repeat injury. Furthermore, effects of these injuries may accumulate as exposure to head trauma increases so patients should be aware of all prior head injuries in order to make informed decisions about their future actions (Guskiewicz et al., 2003). Lastly, undiagnosed concussions can adversely affect future medical management and confound research results (Carroll et al., 2004; Roozenbeek et al., 2013).

### Emerging neuroimaging techniques

Although, conventional MR and CT scans are unable to detect the microscopic damage occurring with DAI, new neuroimaging techniques are emerging that may yield key insight to microstructural changes in the brain following TBI. Diffusion Tensor Imaging (DTI) is a non-invasive MRI method that is sensitive to the microscopic alterations of neuronal tissue, specifically occurring in the white matter (WM) of the brain. DTI relies on the diffusion properties of water to generate measures indicative of the structural integrity of specific WM structures within the brain *in vivo*. For example, movement of water in cerebrospinal fluid (CSF) is unrestricted and, therefore, it is free to diffuse in all directions (isotropic). WM, however, is encapsulated by myelin sheaths, neurofilaments, microtubules, and axonal membranes that restrict diffusion to the direction in which the axon is oriented (anistropic). The two primary indexes of DTI are Fractional Anisotropy (FA) and Mean Diffusivity (MD), which measure the directionality and magnitude of diffusion, respectively. Reduced FA and increased MD measures are thought to be indicative of impaired WM structural integrity. Two additional measures generated by DTI are radial diffusivity (RD) and axial diffusivity (AD), which, although still debated, are thought to be indicators of myelin and axonal health, respectively (Wheeler-Klingshott and Cercignani, 2009). Researchers are also interested in examining the functional impairments DAIs may cause to the brain. Functional MRI (fMRI), is capable of observing these effects by utilizing Blood Oxygen Level Dependent (BOLD) measures in specific regions of the brain, either in response to a cognitive challenge (task) or during the resting state.

Many studies are implementing these emerging neuroimaging modalities to examine the structural and functional damage *in vivo* that is consequential to head trauma. Studies utilizing FA, MD, RD, and AD indexes to measure the structural integrity of WM structures throughout the brain consistently show long lasting damage following TBI (Inglese et al., 2005; Salmond et al., 2006; Kraus et al., 2007; Lipton et al., 2008; Miles et al., 2008; Niogi et al., 2008; Little et al., 2010; Cubon et al., 2011; Ljungqvist et al., 2011; Davenport et al., 2012; Taber et al., 2015). Studies have found significant correlations between the extent of WM damage and the severity of TBI (Matsushita et al., 2011), number of TBI (Davenport et al., 2012), and impaired cognitive function (Salmond et al., 2006; Miles et al., 2008; Niogi et al., 2008). Notably, several of these studies showed no brain damage with the conventional MR and CT scans currently used for diagnosis at the time of initial examination. However, it is worth noting that acute studies utilizing DTI immediately following head injury have produced conflicting results (Mayer et al., 2010; Henry et al., 2011). Until the relationship between acute and chronic alterations to the brain's WM following TBI is better understood, it will be difficult to utilize DTI as a diagnostic tool. Additionally, most of the studies utilizing neuroimaging modalities to study TBI are currently only able to observe significant between-group effects of TBI, but refining these methods to accurately characterize damage to brain tissue at the individual level is the topic of active ongoing research.

### Subconcussive head impacts

Although shortcomings related to the clinical diagnosis and definition of TBI are troublesome and need to be addressed, emerging research suggests that a medically documented history of TBI is not necessary to experience the long-term sequelae of head injuries. Accumulating evidence suggests that frequent exposure to subconcussive blows to the head, even in populations with no history of concussions, can substantially hamper neuronal health (Dashnaw et al., 2012). Recently, researchers have attempted to examine the extent to which this accumulative damage in the brain can occur following subconcussive events, which are defined as blows to the head generating enough force to disrupt neuronal integrity without resulting in clinically evident symptoms. Athletes, particularly those participating in contact sports, are being studied for the effects of subconcussive hits. For example, both collegiate and high school football players are known to experience hundreds, if not thousands, of sub-concussive hits in a single season with an average of 652 hits exceeding 15 g of force (Broglio et al., 2009, 2011; Crisco et al., 2011). There is some concern regarding the validity of studies investigating the effects of subconcussive blows since the events are difficult to define objectively. However, data from accelerometers and access to video recordings have helped to mitigate concerns by limiting the reliance on the inaccurate self-report from athletes.

The contention that subconcussive blows can impair neuronal health is supported by studies utilizing DTI and fMRI to investigate the alterations in the brain's structural and functional connectivity *in vivo*. One DTI study reported evidence of soccer players with significantly impaired WM integrity compared to a group of swimmers who experienced no head trauma (Koerte et al., 2012a). These findings were supported by a study that found the frequency with which soccer players make head contact with the ball is significantly related to both WM damage and, in turn, cognitive impairments (Lipton et al., 2013). Longitudinal studies observing athletes over the course of a single season also indicate that frequent exposure to head trauma, even at subconcussive levels, may have deleterious effects on neuronal health. Ice hockey players were shown to exhibit significant decreases in WM integrity over the course of a single season (Koerte et al., 2012b). In college football players, postseason measures of WM integrity were significantly worse than both postseason measures for noncontact athletes and preseason measures for both groups (McAllister et al., 2014). Additionally, this cohort's head impact exposure (measured by a helmet accelerometer) was correlated with a decrease in WM integrity and impaired cognitive function (McAllister et al., 2014). Another study observed similar changes in WM integrity between pre-season and post-season measures, but further reported that much of these deleterious effects persist chronically after 6 months of no-contact rest (Bazarian et al., 2014). We previously reported chronic structural neuronal damage assessed with DTI in recent military veterans (Iraq and Afghanistan) with blast exposure, even in the absence of a clinically evident concussion (Taber et al., 2015).

Functional impairments are also detected in populations with repetitive exposure to subconcussive insults. Two separate studies demonstrate that high school football players with no clinically evident symptoms of concussion had worsened neurocognitive measures and significant changes to neurophysiology as measured by fMRI (Breedlove et al., 2012; Talavage et al., 2014). Interestingly, this functionally impaired group consisted mostly of linemen, who experience head to head contact at subconcussive levels on nearly every play from scrimmage. Another study employed fMRI to examine the chronic effects of this cumulative damage by investigating a cohort former National Football League (NFL) players who were an average age of 53 years old. The results indicate that this clinically normal cohort of former NFL players exhibited functional hypoconnectivity during resting state fMRI and hyperactivation of brain regions during cognitive tasks in fMRI compared to controls (Hampshire et al., 2013). This finding of hyperactivation during mental tasks could be a compensatory mechanism for brain to overcome the structural damage outlined above in separate DTI studies. These functional and structural impairments observed over the course of a single season are unnerving, especially when considering the collective damage that must be occurring over the course of an entire career (either amateur or professional).

It has been demonstrated that both concussive events and frequent exposure to subconcussive insults result in deleterious effects to the brains structural and functional capacity, often resulting in cognitive impairments. These are significant findings on their own, however, it is essential to take this line of research a step further by examining the effect of damage from head trauma on neurodegenerative diseases later in life. It has been postulated that TBI and repetitive subconcussive events deplete the brain's functional capacity that conspires with aging to push the brain below the threshold for dementia and neurodegenerative disease (Lehman et al., 2012; Bigler, 2013; Smith, 2013; Smith et al., 2013). Across the spectrum of neurodegenerative diseases, there is a unique, complex etiology for each of the conditions and future research is required to better understand the extent to which history of head trauma might factor in to the equation. It should be noted, the ambiguity surrounding the diagnosis and definition of TBI confounds results of studies investigating the long-term effects of head injuries related to neurodegenerative diseases. The inaccurate incidence of TBI is a limitation for all studies investigating any potential correlation, but we are hopeful that improved neuroimaging techniques will help address this limitation by providing more accurate means for diagnosis of concussions at the time of injury. Readers should be aware of this current limitation as they review the following discussion on CTE.

## Chronic traumatic encephalopathy

CTE is a progressive neurodegenerative disease derivative of the cumulative pathophysiological damage incurred by head trauma. The first clinical manifestations are insidious, but typically marked by deterioration in attention, concentration and memory that are often accompanied by disorientation, dizziness, headaches, and mood disorders. As the disease progresses, more prominent symptoms begin to manifest including impulsivity, mood lability, impaired judgment, and overt dementia. In later phases of CTE, these symptoms continue to worsen and are accompanied by impaired motor control which can resemble Parkinsonism with bradykinesia, staggered gait, and vertigo (McKee et al., 2009; Stern et al., 2011). Importantly, the clinical progression of CTE is exceedingly difficult to delineate given that, at this time, diagnoses are not able to be made until post-mortem examination. Therefore, the timeline of symptoms are only available from reports collected from family members of the deceased individuals. Symptoms of drug and alcohol abuse, frequently comorbid with CTE due to impaired decision making and impulse control, pose additional clinical challenges with diagnosis (Gavett et al., 2011). Given these limitations, little is known about the progression of CTE, but it appears that symptoms manifest earlier than other neurodegenerative diseases with the average age of onset between 30 and 50 years and the youngest confirmed case of CTE occurred at 18 years (McKee et al., 2010; Baugh et al., 2012).

Before discussing CTE further, it is important to highlight the current state of CTE research that has produced controversy in the field. The methodological limitations specific to case selection bias present in autopsy based studies can obscure the validity of these findings. There are no appropriately matched healthy controls available to perform direct comparative studies and therefore, while there is strong support, definitive conclusions cannot be made about the potential risk of developing CTE from contact sports and head injuries. It should be noted, however, that every pathologically confirmed case of CTE has at least some history of head trauma, though the severity and frequency of their head injury history is variable (Maroon et al., 2015). Additionally, until recently there were no validated neuropathological criteria to define CTE, which is an entirely new limitation that reduces reproducibility across research groups (Gardner et al., 2014). In April 2015, however, the first set of pathologic criteria for CTE were published as a result of the NINDS/NIBIB consensus meeting sponsored by the NIH (National Institute of Neurological Disorders and Stroke, 2015). Thus, the development of standard pathological criteria for this disease took a major step forward.

Though these limitations make it nearly impossible to investigate the incidence of CTE, neuropathologically confirmed cases of CTE have been observed post-mortem in athletes who were former participants of America football, ice hockey, soccer, and wrestling (Matser et al., 1998; McKee et al., 2009, 2014, 2013; Omalu et al., 2005, 2006, 2010a,b, 2011). Cases of CTE have also been verified in military veterans with blast exposure (Omalu et al., 2011; McKee et al., 2013) and those with history of self-injury (Hof et al., 1991) or domestic abuse (Roberts et al., 1990b). Researchers at the VA-BU-SLI Brain Bank published the most extensive study to date documenting the pathology present in the first 85 brains donated by families of deceased athletes and military veterans, 68 of which were found to be positive for CTE. Furthermore, within this sample, 34 of 35 professional football players were positive for CTE, along with 9 of 9 collegiate players and 4 of 4 ice hockey players (McKee et al., 2013). Notably, it is difficult to extrapolate this data and these numbers are not likely to be representative of disease prevalence within these populations since these case series are highly selective, with tissue procurement often preceded by an abnormal event.

It appears that the scientific community is making strong strides to understand the pathology of CTE, but researchers are also keen to determine the major risk factors for developing this condition. There have been cases of CTE after a single TBI, multiple TBIs, and even with no documented TBIs when the patient reported significant exposure to subconcussive insults (McKee et al., 2014). However, the poor correlation between the number of clinically documented concussions and CTE leaves this question open for debate. Nowinski and Cantu assert that CTE is best correlated with “total brain trauma,” which can be defined as a combination of subconcussive and concussive events (Nowinski, 2013). It is still unclear how other factors like gender, age at youngest head injury, and education may influence risk of developing this disease. Other environmental risk factors include exposure to neurotoxins like glyphophosate, which are found in herbicides, and addiction to methamphetamines (Sekine et al., 2008; Morley and Seneff, 2014). There may also be an inherent risk for CTE that is determined by genetic make-up. Specifically, the E4 allele of the *APOE* gene has received the most attention and, though the existing literature is inconclusive, it may be associated with increased risk of CTE (McKee et al., 2009, 2010; Gavett et al., 2011).

### CTE progression

Researchers at Boston University School of Medicine, at the largest CTE research center in the world, have proposed a staging system for the progression of CTE pathology after performing post-mortem examinations on ~85 brains (McKee et al., 2013, 2014). Table 1 outlines the pathology that characterizes this disease according to those proposed stages, which will need further validation given that they are based solely on retrospective clinical information on a relatively small sample size. For a more detailed review on pathology of CTE, see (McKee et al., 2013).

**Table 1:** **Stages of CTE proposed by McKee et al. (2013)**.

	**Gross**	**Tau**	**Other**	**Clinical symptoms**
Stage I	None	NFTs at depths of cerebral sulci of the frontal cortex	TDP-43 inclusions within sub-cortical white matter and fornix	Mostly pre-clinical
				Some insidious symptoms like headache, depression, attention loss
Stage II	Mild enlargement of lateral and 3rd ventricles	NFT in subcortical white mater, medial temporal lobe, brainstem	Rarely amyloid beta found	Pronounced behavioral/personality changes
	Cavum septum pellucidum			Short term memory loss
	Pallor of LC and SN			
Stage III	Reduced brain weight	NFTs present diffusely in frontal, temporal and parietal cortices. Often concentrated around small vessels and depths of sulci	TDP-43 inclusions now present in cerebral cortex, medial temporal lobe, diencephalon, and brainstem	Memory loss, overall cognitive impairments
	Mild frontal/temporal atrophy			Aggression, mood disorders
	Enlargement of lateral and 3rd ventricles	NFTs present in subcortical structures: hippocampus, entorhinal cortex, amygdala, Nucleus Basalis of Meynart, LC, hypothalamus, mammillary bodies, SN, Raphe nuclei.	Aβ in 13% of cases	75% considered cognitively impaired
	Pallor of LC and SN			
	Atrophy to mammillary bodies, thalamus, hypothalamus, thinning of corpus callosum			
		Rarely present in spinal cord and dentate nucleus of cerebellum		
Stage IV	More pronounced reduction in brain weight	Widespread degeneration resulting from severe NFT deposition.	TDP-43 deposition is more widespread and severe	Cognitive impairments
				Severe mood disorders
	More pronounced atrophy to frontal, temporal lobes and anterior thalamus	Greater NFT inclusion of cerebellum and medulla	Neuronal loss in SN	Language difficulties
	2/3 subjects have septal abnormalities	Prominent myelin loss and astrocytosis of WM in cerebral cortex	Severe spongiosus of cerebral cortex and widespread neuronal loss	Visuospatial difficulties
				Gait/motor control impairments.
	Generalized WM atrophy			
	LC and SN now pale			

Abbreviations: Aβ LC, Locus Coeruleus; NFT, Neurofibrillary Tangle; SN, Substantia Nigra; WM, White Matter.

It is important to note that the proposed progression stages based primarily on the findings of one group and there may be variability across research groups. To highlight this disparity, data from the seminal CTE study conducted by Corsellis et al. (1973), which investigated the brains of boxers, was re-examined by Roberts et al. who reported in 1990 that Aβ was present in 95% of the confirmed cases of CTE (Roberts et al., 1990a). This is substantially higher than reported by McKee et al. who conclude that Aβ may be present in CTE, but it is not a required diagnostic feature of the disease. The recent NIH consensus statement reflects this notion Aβ is not a fundamental criteria for CTE confirmation (National Institute of Neurological Disorders and Stroke, 2015).

### Distinguishing pathological features of CTE

It is essential for researchers to compare the pathological findings associated with CTE to other neurodegenerative diseases. There is significant overlap between CTE and other tauopathies, like Alzheimer's disease (AD), which has benefits as well as disadvantages. Knowing that similarities exist between CTE and AD makes it less daunting; there is a plethora of research on the disease processes and diagnostic methods of AD that researchers can use for devising diagnostic methods for CTE. However, it is vital to differentiate CTE from other neurodegenerative diseases so they can be properly diagnosed and treated. The recent consensus statement goes a long way. A team of neuropathologists were required to differentiate between digitized slides from 25 cases of tauopathy including CTE, Alzheimer's disease (AD), Parkinson-dementia complex of Guam, progressive supranuclear palsy, corticobasal degeneration, primary age-related tauopathy, and argyrophilic grain disease (National Institute of Neurological Disorders and Stroke, 2015).

#### Tau

Many neurodegenerative diseases, including CTE, are characterized by the abnormal deposition of insoluble proteins throughout the CNS. Indeed, Tau is the primary protein involved in its pathology, though it should be noted that this pathology is complex and varied. It is important to note that Tau in its normal, microtubule bound state is not harmful. In fact, Tau is ubiquitous in the healthy adult brain as it is the most abundant microtubule-associated protein (MAP). In this state, Tau promotes the assembly and maintenance of microtubules, which are responsible for intracellular transport, axonal morphology, and cell physiology, all of which signify that Tau is fundamental in maintaining neuronal integrity and axoplasmic transport (Tolnay and Probst, 2003; Amadoro et al., 2006; Williams, 2006).

Tau can exist as six isoforms, all of which are generated from a single gene. These six isoforms differ in the number of N-terminus inserts (0N, 1N, or 2N) and by the presence of either three or four C-terminal microtubule binding repeats (3R or 4R). The presence of 3R and 4R Tau is determined by the inclusion or exclusion of exon10 (Schmidt et al., 2001) and in the healthy adult brain there is an equal ratio of 3R and 4R Tau (Goedert, 2004). Proper functioning of Tau depends on the balance of different Tau isoforms, its state of phosphorylation, and its structural integrity. A perturbation of these parameters may cause Tau dysfunction, ultimately resulting in neurodegeneration (Shahani and Brandt, 2002). Tau becomes harmful when it is no longer bound to microtubules, accumulating as insoluble neurofibrillary tangles (NFTs) in cell bodies and dendrites (Goedert, 2004). Conditions characterized by this abnormal accumulation of Tau share the umbrella term tauopathy. Each tauopathy has a unique series of molecular events in their pathogenic cascade resulting in a conformational change to the structure of the protein. This manifests as either an imbalanced ratio of 3R and 4R variants of Tau or in an abnormal phosphorylation state (Williams, 2006).

The exact mechanisms are not entirely understood, but it appears that Tau may have deleterious effects when it becomes hyperphosphorylated, which may play a role in the neurodegeneration of CTE (McKee et al., 2009; Wang et al., 2013). P-Tau has less affinity for binding with MTs leading to its abnormal accumulation. P-Tau also induces disassembly of MTs, which, when coupled with the abnormal accumulation of Tau, causes significant axonal transport inefficiencies and, ultimately, neurodegeneration (Khlistunova et al., 2006; Zilka et al., 2006; Wang et al., 2013). In CTE, the NFTs are predominately expressed in neurons, but they are also present in lower quantities in astrocytes and oligodendrocytes (Yoshiyama et al., 2013).

Though Tau is the hallmark of CTE, it is also involved in the pathology of a number of other disorders including Progressive Supranuclear Palsy, Pick's disease, Corticobasal degeneration, Frontotemporal dementia, and most notably in Alzheimer's disease. However, these tauopathies differ in topographic distribution of tau, isoform profile, and phosphorylation state (Williams, 2006) with some being more similar than others. Among these diseases, CTE is most similar to AD based on their respective Tau profiles (Schmidt et al., 2001).

Yet, key differences exist between the histopathology of CTE and AD. Most of the distinguishing characteristics of CTE's Tau profile are related to the spatial distribution of NFTs. NFT distribution in CTE is sporadic, while AD shows a common and highly consistent pattern of distribution (Braak and Braak, 1991; McKee et al., 2009). The perivascular clustering of NFTs and the prominent presence of NFTS at the depths of sulci are also unique to CTE (McKee et al., 2013). Greater NFT involvement in the superior and dorsolateral frontal lobes is present in many cases of CTE, particularly involving football players (McKee et al., 2014). Interestingly, this Tau distribution in CTE may match the biomechanics of head injuries since there is speculation that the depths of the sulci are areas of stress concentration for shear forces given that athletes of contact sports experience frequent high-intensity impacts on the crown of their helmets, which corresponds to pathological findings in the superior and dorsal-lateral frontal lobes (Hof et al., 1992; Cloots et al., 2013; McKee et al., 2014). Notably, this region is also one in which functional impairments were observed via fMRI in a group of football players (Talavage et al., 2014). Tau histopathology also demonstrates the presence of astrocytic tangles in CTE that are entirely absent from AD (McKee et al., 2013). Lastly, there is significantly more WM involvement in CTE than in AD since axons are relatively spared in AD (McKee et al., 2013), which could be related to the DAI from concussions and repetitive subconcussive insults (Johnson et al., 2013b).

#### Amyloid-beta

Another histopathological difference between CTE and AD is the presence and distribution of amyloid-Beta (Aβ) (Braak and Braak, 1991). Though, the extent of Aβ pathology is highly variable, the relative paucity of Aβ in CTE may be used to help differentiate the condition from Alzheimer's disease. Samples from the VA-BU-SLI Brain Bank showed Aβ in only 27% of pure CTE cases and in 44% of all CTE cases (including those with comorbid neurodegenerative diseases) (McKee et al., 2013). Notably, the cohort of CTE subjects with Aβ were significantly older than those without Aβ in this study. In this subset of CTE positive brains, the Aβ was present in the form of diffuse plaques, neuritic plaques, and vascular plaques (McKee et al., 2013). More recently, a study investigating the role of Aβ in CTE found abnormal amyloid deposition in 52% of 114 CTE patients during post-mortem examination (Stein et al., 2015). This study also attempts to delineate potential reasons for CTE cases with Aβ deposition as opposed to those without, while also examining the potential effects this Aβ pathology may have on other aspects of the disease. Among the subjects with CTE, this study reports (1) mean age at time of symptom onset and death were both higher in CTE subjects with Aβ, (2) Aβ deposition was significantly associated with presence of APOE e4 allele, (3) Aβ deposition was correlated more significant Tau deposition independent of age, and (4) Aβ presence was a strong predictor for both Lewy-Body co-morbidity and dementia independent of age (Stein et al., 2015). A comparative analysis was also performed with non-selected community-based autopsy series of 2332 subjects without CTE, which showed that the odds of developing Aβ plaque pathology were 3.8 times higher in CTE than in normal aging (Stein et al., 2015).

Additionally, the presence of Aβ is not a binary feature; its regional distribution is significant in distinguishing between CTE and other neurodegenerative diseases. For example, according the NIH consensus statement for diagnostic criteria of CTE, the neuropathologists contend that neurofibrillary degeneration in the hippocampus caused by Aβ plaques is an exclusion criteria for diagnosis of CTE (National Institute of Neurological Disorders and Stroke, 2015). Another key distinction between the Aβ pathology of CTE and AD is the sulcal predilection of Aβ in CTE. Researchers have found there was no difference in sulcal vs. gyral Aβ plaque deposition in subjects with only AD, but subjects with CTE had significantly greater Aβ plaque burden in the depths of the sulci (Stein et al., 2015). This could be explained by greater concentrations of stress at the sulcal depths caused by the acceleration-deceleration forces in head injuries (Cloots et al., 2008).

#### Other

Lewy body and TDP-43 proteinopathy can also coexist with Tau pathology in CTE, which are the hallmark proteins for the pathology of Parkinson's disease and Amyotrophic Lateral Sclerosis (ALS), respectively. Of the CTE cases identified at the VA-BU-SLI Brain Bank, 22% had Lewy Pathology (McKee et al., 2013). TDP-43, however, may be much more common in CTE. One study found that TDP-43 proteinopathy coexisted with tauopathy in 80% of their CTE cases (McKee et al., 2010). Those with TDP-43 pathology are thought to exhibit a subset of CTE, with motor symptoms progressing at a faster rate in a manner similar to ALS (McKee et al., 2014). These cases are classified as CTE-MND, which denotes the comorbid motor neuron disease.

## Potential detection of CTE pathology with PET scans

Positron Emission Tomography (PET) facilitates our understanding of both normal and abnormal brain function on a molecular level in real time. It is a powerful molecular imaging modality that can deliver quantitative measures of various metabolic processes in the brain with targeted radiopharmaceuticals (Pike, 2009). These radiotracers emit positrons, which then undergo radioactive decay before colliding with electrons to produce 2 photons. The PET scanners are capable of detecting these photons (gamma rays) in order to reconstruct an image of spatial density indicative of functional activity and metabolic processes within the brain (Portnow et al., 2013). The utility of PET scans is highly adaptable and its function is contingent on the specific biological process in question. A large number of neurometabolic processes, that are often pathological in nature, can be targeted using specifically developed radioactive tracers.

As the name suggests, these radiopharmaceuticals are usually labeled with one of the following short-lived positron-emitters: Carbon-11 (^11^C) or Fluorine-18 (^18^F) (Pike, 2009). Brain PET probes typically have one of two different mechanisms of action depending on the process they were developed to examine. The first class radiopharmaceuticals are typically analogs of endogenous compounds that are designed to enter the brain via active transport before accumulating upon metabolic transformation in the brain. The textbook example of such a probe is 2-[^18^F]fluoro-2-deoxy-D-glucose (FDG), an analog of glucose, which can be utilized to measure metabolism in the brain where glucose is the sole energy source (Pike, 2009; Gandy et al., 2014). Alternatively, the other class of radioactive pharmaceuticals is characterized by having no such recognized transport system and must bind to a specific protein target, allowing for the detection and quantification of proteins linked to disease (Pike, 2009). The most well-known example is Pittsburgh Compound B (PiB), a tracer that provides quantitative information on Aβ deposition in living human brains (Klunk et al., 2004). There are now multiple FDA approved amyloid tracers that are utilized clinically as well as in research.

### Imaging amyloid-beta

The PiB compound, initially developed to track amyloid deposition in Alzheimer's disease, has also been utilized to study Aβ pathology following head trauma. Aβ plaques, a hallmark of AD, can appear hours within head injury in all age groups and are found in up to 30% of patients who die acutely following TBI (Roberts et al., 1991, 1994; Graham et al., 1995; Ikonomovic et al., 2004). The deposition of these Aβ plaques, representative of AD, can occur during “normal aging,” but this process appears to be accelerated by head injuries (Johnson et al., 2012). Widespread Aβ deposition increases in cortical gray matter and striatum of TBI patients (median age of 35 years) between 1 and 361 days following the injury (Hong et al., 2014). Targeting these plaques with specific radioactive probes via PET can answer key unknowns regarding the temporal profile of Aβ deposition following TBI and the subsequent clinical symptomology.

Another PET ligand specific to Aβ, ^18^F-Florbetapir, is also being used to investigate the mechanisms of amyloid deposition following TBI. Using this tracer, one study attempted to examine the effects of Aβ on cognitive impairment following mTBI by comparing three cohorts: healthy controls, mTBI with cognitive impairment, and mTBI with no cognitive impairment. The PET scans for this study occurred an average of 6 years post-injury. The results indicate there is significantly greater amyloid deposition in the mTBI patients exhibiting cognitive dysfunction, but the cognitively normal mTBI does not significantly differ from the healthy controls (Yang et al., 2015). These results either suggest that the cognitively normal mTBI group never accumulated Aβ following the injury or that they had stronger neuro protective response that resulted in Aβ clearance following the head trauma. Future longitudinal studies are required to better investigate the role of Aβ in both the acute and chronic phases of recovery from head trauma.

### Imaging of neuroinflammation

It is speculated that the neuroinflammatory processes following head trauma have deleterious effects over time that may relate to the pathology characteristic of CTE (Cunningham et al., 2005; Whitney et al., 2009; Brown and Neher, 2010; Blaylock and Maroon, 2011). Radiotracers like [^11^C](R)PK11195 (PK), which quantitatively measures the presence of activated microglia, are now being utilized to examine this response *in vivo*. One PET study employing this PK ligand indicates a chronic, perpetual neuroinflammatory response in TBI patients with significantly increased microglial activation up to 17 years post injury (Ramlackhansingh et al., 2011). Further, this finding is substantiated by a separate immunohistochemistry study reporting elevated microglial activation up to 18 years following a single head injury (Johnson et al., 2013a). As Blaylock and Maroon posit, this perpetual inflammatory response could be the underlying mechanism responsible for triggering the hyperphosphorylation of Tau and the pathological cascade that follows (Blaylock and Maroon, 2011).

### Developing a probe for tau

Researchers should be able to do the same for Tau, specifically the P-Tau that is a hallmark of CTE, upon the development of an effective radiopharmaceutical. As of this article's publication, a reliable radiotracer to track Tau deposition *in vivo* is still under development due to a number of challenging criteria. In order for the radioactive probes to be suitable for the quantitative imaging of protein targets in the human brain, they must exhibit: (1) high affinity for target, (2) high selectivity for target, (3) high permeability of blood-brain barrier, (4) low affinity for non-specific binding, (5) absence of confounding radiometabolites, and (6) suitable pharmacokinetics in relation to half-life, brain uptake, and washout kinetics (Pike, 2009). The P-Tau present in CTE presents added unique challenges as a target for radioactive tracers. First, the P-Tau aggregates are primarily intracellular, although it is also found extracellularly upon neuronal death (Jensen et al., 2011; Fodero-Tavoletti et al., 2012). This implies that the radiotracer must also be permeable to cell membrane in addition to blood brain barrier (BBB) in order to bind to P-Tau, which imposes new constraints on the tracer's size and lipophilicity. Second, the structure and morphology of Tau is highly variable making it a much more complex target than Aβ and other proteins (Jensen et al., 2011; Fodero-Tavoletti et al., 2012). To further complicate the matter, Tau aggregates are subject to an array of posttranslational modifications including acetylation, glycosylation, glycation, prolyl-isomerization, nitration, and ubiquitination, all of which have the potential to alter the morphology of Tau and, thus, affect the ability of specific tracers to bind to it as a target (Fodero-Tavoletti et al., 2012; Villemagne and Okamura, 2014). Third, P-Tau shares the same beta-sheet secondary structure as Aβ, which presents special challenges to developing a radiotracer that selectively binds to P-Tau (Fodero-Tavoletti et al., 2012).

Scientists are attempting to identify a probe that fits all of these criteria for the quantitative imaging of P-Tau aggregation. In 2005, Okamura and colleagues screened ~2000 small molecules (primarily quinolone and benimidazole derivatives) in an attempt to find a fitting candidate (Okamura et al., 2005). In the years since, several of these compounds and others showing the greatest potential as radiotracers for P-Tau have been serially analyzed *in vitro, ex vivo*, and *in vivo* in animal models. The compounds that survive the gauntlet of tests with promising results have the potential to be approved for human trials. The following section summarizes select probes with the greatest potential.

#### FDDNP

The compound 2-(1-(6-[(2-[fluorine-18]fluoroethyl)(methyl)amino]-2-naphthyl)- ethylidene)malononitrile (FDDNP), developed in 2002, was the first PET imaging probe capable of targeting P-Tau. FDDNP showed such promising results in early *in vitro* studies that it was deemed suitable to human trials *in vivo* to detect P-Tau (Shoghi-Jadid et al., 2002; Agdeppa et al., 2003). It was the first radiotracer capable of quantitatively measuring the NFTs of P-Tau *in vivo*, but it is not specific to P-Tau. FDNNP also binds to Aβ, which means PET binding may indicate either the NFTs of P-Tau or Aβ plaque deposits (Small et al., 2006; Yaqub et al., 2009; Shin et al., 2011). It is unable to distinguish between Tau and Aβ aggregation, but researchers can make up for this lack of specificity by combining values from FDNNP PET with our pre-existing knowledge of regional pattern of Tau and Aβ deposition observed in various diseases. Additional PET scans can also be performed with a radiopharmaceutical specific to Aβ, which researchers can then subtract from FDNNP scan to differentiate between P-Tau and Aβ.

Most recently, FDDNP PET was employed to measure Aβ/Tau deposition in retired NFL players with histories of cognitive and/or mood symptoms; a group with significant exposure to head trauma and is at greater risk of CTE. Though the data is limited by a small sample size, the results from this study show that FDDNP binding values are significantly greater for former players in subcortical structures that match the P-Tau deposition patterns observed in post-mortem examinations of CTE (Table 1). Furthermore, the FDDNP binding values increase with the extent of the subject's concussion history indicating a correlation with exposure to head trauma (Small et al., 2013).

#### ^18^F-THK523

Quinoline and benzimidazole derivatives were identified as candidates for Tau imaging tracers by screening small molecules capable of binding to β-sheets resembling the structure of Tau. THK523, a quinolone derivative, was tagged and radiolabeled with ^18^F to be used as a PET probe. In 2011, the utility of ^18^F-labeled THK-523 (^18^F-6-(2-fluoroethoxy)-2-(4-aminophenyl)quinoline) as a radiotracer was tested in a series of *in vitro, ex vivo*, and *in vivo* experiments in mice. With the results from these tests, researchers determined that ^18^F-THK523 has high selectivity for P-Tau with an overall 5-fold increase for binding over Aβ with no binding to α-synuclein. Additionally, the probe was small enough and adequately lipophilic to penetrate the BBB and cell membrane, while also exhibiting significantly greater retention in Tau transgenic mice (Fodero-Tavoletti et al., 2011). Another group of researchers further examined the selectivity of ^18^F-THK523 using human brain tissue from deceased patients of Alzheimer's disease, Parkinson's disease, and several Taupathies (Pick's disease, Progressive Supranuclear Palsy, Corticobasal Degeneration). The results indicate that ^18^F-THK523 has high selectivity for the P-Tau found in CTE with significantly lower selectivity for Aβ, Lewy bodies, and other Tau variants (Fodero-Tavoletti et al., 2014). The original developers claim a possible limitation of this probe is poor pharmacokinetics (slow brain uptake/clearance) based on their preclinical evaluations (Okamura et al., 2013).

Another group of researchers performed a human clinical trial to test the utility of ^18^F-THK523 in an AD population (Villemagne et al., 2014). As expected from pre-clinical experiments, ^18^F-THK523 exhibited strong affinity and selectivity for Tau *in vivo*. Global cortical ^18^F-THK523 binding provided a robust distinction between AD patients and healthy controls. Furthermore, the level of ^18^F-THK523 binding was significantly correlated with cognitive parameters. A separate PiB PET scan was employed to demonstrate a marked contrast in its regional distribution compared to ^18^F-THK523, and thus a significant distinction between P-Tau and Aβ pathology. The researchers also included group of semantic dementia patients, a disease marked by presence of TDP-43, all of which showed no binding for ^18^F-THK523 or PiB (Villemagne et al., 2014). Interestingly, the results from this clinical trial indicate adequate brain uptake and clearance to contradict the assertion that this probe displays poor pharmacokinetics (Okamura et al., 2013). However, this study highlighted one key limitation that will preclude ^18^F-THK523 from becoming a capable radiotracer for Tau: ^18^F-THK523 exhibited extremely high white matter retention. This high nonspecific binding prohibits simple visual inspection and a great deal of quantitative analysis (Villemagne et al., 2014).

#### ^18^F-THK5105 and ^18^F-THK5117

Using ^18^F-THK523 as a platform, the original developers continued compound optimization to develop two novel compounds within the same family of ^18^F-labeled arylquinoline derivatives: ^18^F-THK5105 and ^18^F-THK5117 (Okamura et al., 2013). *In vitro* binding assays indicate that both THK-5117 and THK-5105 have higher binding affinity for P-Tau than their predecessor, THK-523 (Okamura et al., 2013). This was further substantiated by additional experiments in which both compounds had high binding affinities in specific regions of AD brain sections that follow the known distribution of Tau deposition (Okamura et al., 2013). THK5117 and THK5105 also exhibit low nonspecific binding as the binding affinity was determined to be 25 times lower for Aβ plaques. Additionally, these newly developed compounds exhibited greatly improved pharmacokinetics in mice with faster brain uptake and more rapid clearance as compared with THK-523 (Okamura et al., 2013). Given its compatibility with the established criteria for potential PET radiotracers, the researchers recently selected one of these novel compounds for a human clinical trial.

^18^F-THK5105 and ^11^C-PiB were both employed to investigate P-Tau and Aβ pathology, respectively, in a PET study of Alzheimer's disease patients and age-matched healthy controls (Okamura et al., 2014). The novel ^18^F-THK5105 successfully differentiated AD patients as the radiotracers displayed high levels of binding in regions that matched the known distribution of Tau. Additionally, there was high selectivity for Tau with no ^18^F-THK5105 binding to Aβ, which was further validated by the subsequent ^11^C-PiB scan. Additionally, the level of ^18^F-THK5105 binding was significantly correlated with the severity of dementia. Another interesting finding from this study indicates that hippocampal atrophy is strongly correlated with ^18^F-THK5105 retention, but not ^11^C-PiB. ^18^F-THK5105 displayed pharmacokinetic properties that are superior to all other known tau radiotracers (THK523, T807, T808, and ^11^C-PBB3) giving it a unique advantage. Compared with THK-523, this novel radiotracer showed improved levels of nonspecific binding with less WM binding in cortical and subcortical areas. However, unfavorable non-specific WM retention is still present especially within the brainstem and thalamus (Okamura et al., 2014).

As a final note, the researchers assert they are optimistic that they will observe improved pharmacokinetics and enhanced signal-to-background ratio against WM with ^18^F-THK5117 based on their findings from pre-clinical experiments (Okamura et al., 2014). Clinical trials with this compound are currently ongoing.

#### ^18^F-T807 and ^18^F-T808

In the initial screening of compounds, two novel benzimidazole pyrimidine derivatives were identified for their ability to bind to P-Tau and labeled with ^18^F to create two potential PET probes for tauopathy: ^18^F-T807 and ^18^F-T808 (Shoup et al., 2013). After *in vitro* assays with human brain tissue and *in vivo* experiments with mice, these novel compounds satisfied all major criteria for PET radiotracers demonstrating the ability to permeate BBB & cell membrane, favorable pharmacokinetics, high selectivity & affinity for P-Tau, and absence of radiometabolites (Zhang et al., 2012; Xia et al., 2013). Specifically, they exhibited selectivity that was >27 fold greater for P-Tau over Aβ plaque. Preclinical studies of these novel compounds also demonstrated minimal nonspecific white matter binding, which will hopefully translate to greater clinical utility compared to the previously discussed radiotracers (Chien et al., 2013).

The first clinical trial employing ^18^F-T807 was performed on six human subjects with different cognitive profiles (3 healthy, 2 AD, 1 MCI). ^18^F-T807 was significantly higher for both patient populations compared to the controls and, further, the binding levels were correlated with cognitive function. Additionally, the pattern of tracer retention overlaps with the known areas of P-Tau formation according to Braak staging in this patient population with progressing levels of dementia (1 MCI, 1 mild AD, 1 severe AD). This proposed tracer also displayed exquisite selectivity for P-tau over Aβ (>29 fold) *in vivo*. Significantly, unlike other ^18^F PET probes,^18^F-T807 exhibited very low non-specific binding in both the gray matter and white matter of the brain yielding improved contrast and, thus, greater clinical utility (Chien et al., 2013). These are promising results, but it is worth noting that this is an incredibly small sample size. Further, studies are required to validate these findings and, at the time of publication, there is a large scale clinical trial investigating the utility of T807 as a Tau tracer.

The same group of researchers went on to perform the first clinical trial with ^18^F-T808 shortly after with 11 human subjects (8 AD, 3 healthy controls). Recall, this compound showed similar Tau affinity and selectivity as ^18^F-T807 in preclinical studies, but it also displayed faster pharmacokinetics in rodents. The clinical trial corroborated these findings. Researchers were able to successfully differentiate between AD patients and healthy controls because of significantly greater ^18^F-T808 uptake in areas that mirror the known regions of P-Tau distribution (Chien et al., 2014). Due to the death of one subject 2 weeks after the PET scan (unrelated to procedure), an opportunity arose to compare an exploratory post mortem pathological analysis of the brain tissue to the ^18^F-T808 PET scan acquired 2 weeks prior. In this unique case, the regional distribution and levels of ^18^F-T808 binding were consistent with the pathological pattern observed during the autopsy (Kolb et al., 2013). Similar, to ^18^F-T807, ^18^F-T808 also resulted in low tracer retention in white matter. This yields an improved contrast that may help clinicians and researchers detect smaller amounts of tau pathology in the brain. Additionally, the pharmacokinetics were enhanced compared to ^18^F-T807, with ^18^F-T808 exhibiting more rapid uptake, distribution, and clearance. However, ^18^F-T808 does have one key limitation—it exhibits greater levels of ^18^F-defluorination, which results in excessive bone uptake of the tracer and exposure to radiation (Chien et al., 2014).

#### ^11^C-PBB3

This PET probe is unlike the rest. It is derived from a separate class of Tau ligands, phenyl/pyridinyl-butadienyl-benzothiazoles/benzothiazoliums (PBBs), and it is tagged with ^11^C as opposed to ^18^F (Maruyama et al., 2013). There are advantages and disadvantages associated with each of these radiolabels. ^18^F radiolabels are typically preferred due to their convenience. They have a longer half-life (110 min) and can be synthesized at a central production center before being distributed to the study site(s). ^11^C has a much shorter half-life (20 min) that precludes this regional distribution. However, the ^11^C radiolabel permits two or more serial scans to be conducted on the same subject with different radiopharmaceuticals on the same day just several hours apart (Mathis and Klunk, 2013). This is beneficial for researchers and clinicians interested in performing consecutive PET scans with ^11^C-PBB3 and ^11^C-PiB.

Several PBB candidates were exposed to thorough preclinical evaluation and PBB3 was the compound that best fulfilled the criteria for radiopharmaceuticals. As such, a clinical trial was developed to test its utility as a diagnostic marker for tauopathies in a study with six adult subjects (3 AD, 3 Healthy). The results were promising. There were distinct binding patterns for both ^11^C-PBB3 and ^11^C-PiB that match the expected P-Tau and Aβ pathology, respectively. There was a correlation between ^11^C-PBB3 binding and cognitive deficits that was absent with the ^11^C-Pib data. The ^11^C-PBB3 exhibited adequate contrast with limited nonspecific binding to white matter, however, it accumulated in the dural venous sinuses for both the AD patients and the controls (Maruyama et al., 2013). An ancillary finding resulted from additional ^11^C-PBB3 and ^11^C-PiB PET scans of a subject who was clinically diagnosed with corticobasal degeneration. For this subject, ^11^C-PiB stayed at the control level, but there was increased retention of ^11^C-PBB3 in the basal ganglia. This indicates that ^11^C-PBB3 is specific to Tau, but it might bind to conformations other than what is expected in CTE and AD populations (Maruyama et al., 2013).

## Discussion and future directions

There is tremendous potential clinical utility in both detecting and quantifying changes of P-Tau aggregation. When a reliable and effective radiopharmaceutical is available for P-Tau, PET will be a vital tool for researchers to uncover the underlying pathological processes of CTE. At risk populations, like athletes of contact sports, should be tracked longitudinally to improve our understanding of disease mechanisms even prior to exhibiting signs and symptoms of CTE. Pre-clinical phenotypes might exist that could be identified to treat at-risk populations before their underlying pathology progresses to CTE. Further, symptoms of individuals presenting with the signs of CTE can be observed over time as a function of Tau pathology.

Rather than solely relying on PET, longitudinal studies should also employ multimodal neuroimaging techniques. PET scans will provide terrific insight into the disease mechanisms and pathogenic cascade, but for several reasons it is unlikely to be the long term answer for clinical diagnosis. Unlike MRI, PET is invasive and associated with greater risks stemming from radiation exposure. Radiopharmaceuticals are not only expensive, but also difficult to synthesize and transport in timely fashion. Once a reliable and effective Tau radiopharmaceutical is developed, it would be best to use PET in conjunction with other MRI modalities. When used in combination, the findings from PET scans may be strongly correlated with structural MRI, DTI, and fMRI results to the extent where entirely new biomarkers will be established for CTE that solely rely on MRI modalities.

It is also important to build on our current understanding of CTE by more appropriately employing the technologies that are already available to us. For example, the majority of DTI studies investigating the effects of concussions are done 0–6 months post injury. They are fairly consistent in showing impaired WM integrity with conflicting findings primarily resulting from studies of the hyperacute response to head trauma just 1–5 days after injury (Niogi and Mukherjee, 2010). The few studies utilizing DTI to investigate the chronic effects of head trauma >2 years post-injury report significantly impaired WM integrity that correlates with cognitive impairment (Kraus et al., 2007; Geary et al., 2010; Little et al., 2010). It is plausible that much of the damage done to the axons occurs secondarily to the initial injury through complex biological mechanisms that may not be observable in the early phases of recovery (Morley and Seneff, 2014). It is important for future studies to observe those with frequent exposure to head trauma longitudinally, even after exposure to head trauma has ended. The initial insult of the injury(s) may incur perpetual neuronal damage due to the underlying immunoexcitatory response many years after the last subconcussive insult, which has be documented 18 years after the initial head injury (Ramlackhansingh et al., 2011; Johnson et al., 2013a). If this is the case, then clinicians may have the potential to stop ongoing neuronal damage from progressing to a full blown neurodegenerative disease. This has already been demonstrated in mice: the reduction of NFTs and Aβ levels (but not Aβ alone) resulted in improved neuronal health while also ameliorating cognitive decline and improving motor function (Oddo et al., 2006).

For this reason, another immediate application of this PET technology will be quantitatively measuring the effectiveness and utility of drugs capable of targeting NFTs. If this Tau mediated neurodegeneration is detected and properly treated, cognitive function and overall mental health can potentially be restored. There are several therapeutic strategies to treat Tau mediated neurodegeneration that are currently being explored.

## Conclusion

There is mounting evidence that TBI or significant exposure to repetitive subconcussive insults (or any combination of the two) may trigger a pathological cascade that results in abnormal accumulation of P-Tau, which may ultimately manifest as CTE. At this time, however, there are many limitations that must be addressed to better understand this disease. First, brain banks like the VA-BU-SLI investigating CTE are conducting studies on biased samples that lack low-exposure subjects, making it difficult to extrapolate incidence rates. Second, there is currently no way to diagnose CTE *in vivo*, which prevents tracking symptoms as they progress. Third, because awareness is still relatively low and symptoms overlap with other disorders (i.e., AD, ALS), CTE can easily be misdiagnosed. Fourth, inaccurate medical records of concussion history based on ambiguous definitions and diagnostic criteria, makes the correlation between head injuries and neurodegenerative diseases difficult to assess retrospectively. Fifth, there is a large knowledge gap in non-head trauma risk factors that may make individuals more susceptible to CTE. Despite these limitations, the existing scientific evidence increasingly supports that exposure to high levels of “total brain trauma” contributes to the unique pathology observed in CTE. Nevertheless, we need to be honest and forthcoming with these limitations. With the recent high-profile cases of CTE, the media has begun to sensationalize this disease, which means researchers and clinicians need to proceed with caution, particularly when counseling patients with significant exposure to head trauma.

The gaps in knowledge created by these limitations are rapidly diminishing and will continue to do so. The most pressing issue, however, is developing an *in vivo* diagnostic tool capable of detecting CTE. Such a tool should be used in conjunction with other, more readily available, neuroimaging modalities (e.g., MRI) to establish an additional stand-alone biomarker for CTE. With a reliable *in vivo* diagnostic tool, there would be a more accurate prediction of disease prevalence and we could, for the first time, track the progression of disease pathology and symptomology through longitudinal studies. Additionally, it will be vital for clinicians to differentiate between CTE and other neurodegenerative diseases, particularly since mortality from neurodegenerative diseases is significantly greater in populations with high levels of “total brain trauma.” Improved differential diagnosis will increase the likelihood that patients receive the appropriate treatment. Lastly, such a tool to would rapidly accelerate our ability to design therapeutic strategies capable of preventing and treating this debilitating disease.

### Conflict of interest statement

P. Murali Doraiswamy has received research grants from Avid/Lilly, Elan, Bristol-Myers, Ono, Sanofi, Novartis, Medivation and Neuronetrix in the recent past. He has received advisory or speaking fees from the University of California, National University of Singapore, University of Cambridge, Royal College of Psychiatrists, Radiologic Society of North America, Alzheimer's Association, Alzheimer's Foundation of America, Postgraduate Press, Avid/Lilly, Medivation, Bristol Myers, Accera, Piramal, Grifols, Baxter, Nutricia, Great Falls Living, Sonexa, Schering, TauRx, Baxter, Elan, Genomind, Shire, Neuroptix, Bayer, Neuronetrix, Otsuka, AstraZeneca, Envivo, Targacept, Abbvie, Neurocog Trials, Lundbeck and Edwards Hospital. Rajendra A. Morey and Mark Sundman declare that the research was conducted in the absence of any commercial or financial relationships that could be construed as a potential conflict of interest.
